# MRI of the axial skeleton in spondyloarthritis: the many faces of new bone formation

**DOI:** 10.1186/s13244-019-0752-4

**Published:** 2019-07-24

**Authors:** Frederiek Laloo, Nele Herregods, Jacob L. Jaremko, Philippe Carron, Dirk Elewaut, Filip Van den Bosch, Koenraad Verstraete, Lennart Jans

**Affiliations:** 1 Department of Radiology and Medical Imaging, Ghent University Hospital, Corneel Heymanslaan 10, 9000 Ghent, Belgium; 2 Department of Radiology & Diagnostic Imaging, University of Alberta Hospital, 8440-112 Street, Edmonton, Alberta T6G 2B7 Canada; 3 Department of Rheumatology, Ghent University Hospital, Corneel Heymanslaan 10, 9000 Ghent, Belgium; 4 VIB Inflammation Research Center, Unit for Molecular Immunology and Inflammation, Ghent University, Technologiepark 927, 9052 Ghent, Belgium

**Keywords:** Magnetic resonance imaging, Spondyloarthritis, Spine, Sacroiliac joint, Ankylosis

## Abstract

Spondyloarthritis has two hallmark features: active inflammation and structural lesions with new bone formation. MRI is well suited to assess active inflammation, but there is increasing interest in the role of structural lesions at MRI. Recent MRI studies have examined the established features of new bone formation and demonstrated some novel features which show diagnostic value and might even have potential as possible markers of disease progression. Although MRI is not the first imaging modality that comes into mind for assessment of bony changes, these features of new bone formation can be detected on MRI—if one knows how to recognize them. This review illustrates the MRI features of new bone formation and addresses possible pitfalls.

## Key points

- New bone formation is a hallmark feature of spondyloarthritis.
- New bone formation can be reliably assessed on MRI.
- MRI shows new bone formation within the sacroiliac joints.
- MRI shows (peri)-discal new bone formation in the spine.
- The facet joints and manubriosternal joint show new bone formation as well.

## Background

Spondyloarthritis (SpA) represents a group of inflammatory rheumatic diseases, inter-related by clinical, genetic, radiological, and therapeutic characteristics. Axial SpA manifests as arthritis and enthesitis of the axial skeleton, clinically associated with inflammatory back pain [1–5]. As the disease progresses, new bone formation becomes a prominent feature in the axial skeleton—resulting in reduced mobility, deformity of the spine, and increased morbidity [1, 2, 6]. Both active inflammation and new bone formation are considered hallmark features of SpA [1, 2, 7–10].

Establishing a diagnosis for SpA is based on the combined presence of a number of clinical features combined with imaging. As such, imaging of the axial skeleton has attained an important role in diagnosis, classification, and follow-up of SpA [11]. For initial evaluation of axial SpA, MRI of the sacroiliac joints is the preferred technique [11, 12]. For evaluation of established disease, both MRI and radiography are considered useful: radiography to detect structural bony changes and MRI of the axial skeleton to monitor inflammation and structural lesions and to evaluate treatment [13, 14].

In current clinical practice, when evaluating an MRI for features of SpA, the radiologist will often focus on the inflammatory lesions. Correspondingly, the definition of a “positive MRI” in the Assessment of SpondyloArthritis international Society (ASAS) classification criteria focusses on bone marrow edema and does not include structural lesions [11, 12]. Nevertheless, as stated in the ASAS classification criteria, bone marrow edema should only lead to a “positive MRI” if it is “suggestive of SpA” [11]. This addition to the definition implies the need of a certain qualitative aspect, which might be established by including early signs of structural lesions in the MRI assessment.

Recently, several studies have examined features of new bone formation on MRI of the axial skeleton in patients with SpA and found features that showed potential as diagnostic features or as markers of disease progression [15–21]. The inclusion of these and other features of structural lesions might provide a useful additional qualitative aspect to MRI assessment for SpA.

The aim of this pictorial review is to familiarize the reader with the features of new bone formation on MRI of the axial skeleton in axial SpA and to point out possible pitfalls in interpretation.

## MRI features of new bone formation in the sacroiliac joints

**Fig. 1:**
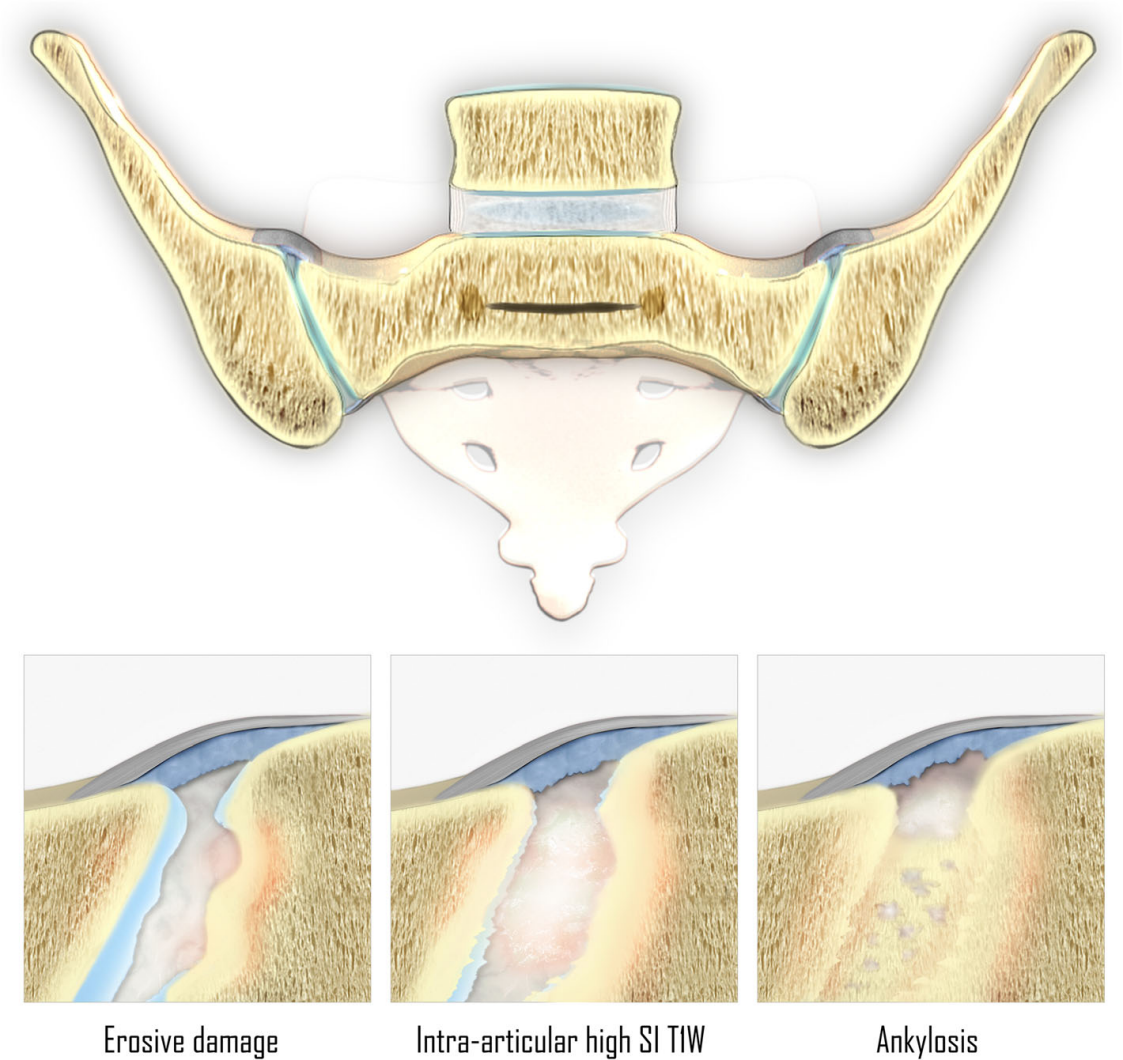
Features associated with new bone formation in the sacroiliac joints of patients with SpA. Note: erosive damage occurs before new bone formation. (SI T1W = signal intensity on T1-weighted MR images)

An illustration of the features associated with new bone formation in the sacroiliac joints has been presented in Fig. 1.

### Intra-articular high signal intensity on T1-weighted MR images

**Fig. 2:**
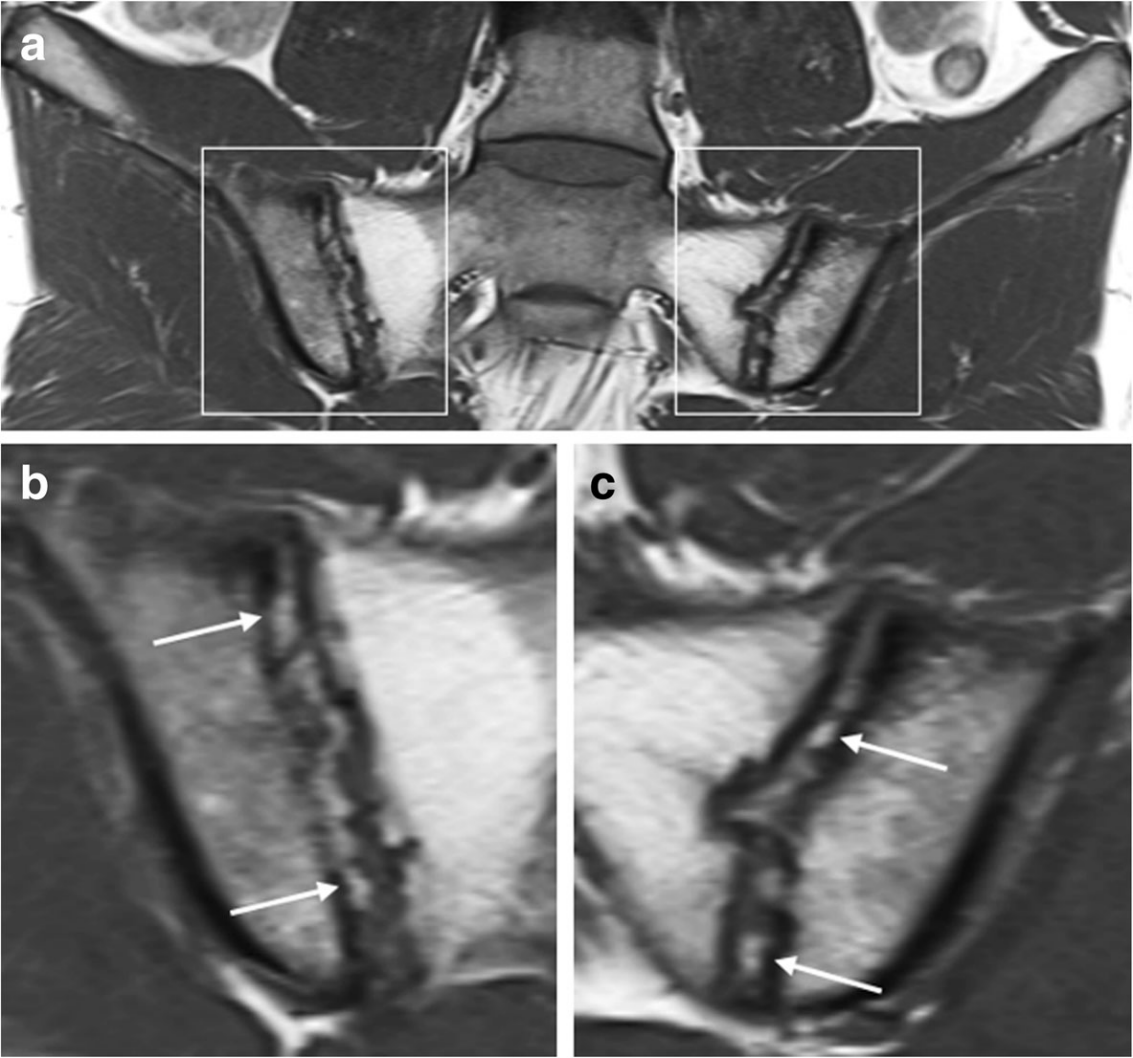
Intra-articular high signal intensity in the sacroiliac joints space on T1-weighted MR images. Coronal oblique T1-weighted MR images acquired in a 25-year-old man with SpA show high intra-articular signal intensity (arrows). This high signal intensity is clearly filling up the eroded iliac bone on the right (i.e., “backfill”). Extensive fatty degeneration of the bone marrow of the sacral side of the sacroiliac joints is present, i.e., post-inflammatory structural changes. Note: **b** and **c** are enlargements of the regions outlined in **a**

“Backfill” (Fig. 2) has been defined as the presence of high signal intensity—similar to that of adipose tissue—on T1-weighted MR images within the sacroiliac joint space, present on two consecutive slices and measuring 10 mm or more parallel to the subchondral bone plate on at least one slice [19, 20]. This has been hypothesized to represent metaplastic tissue refilling the eroded subchondral bone [15]. However, no consensus exists on what this MRI feature really represents, as no histopathological analysis of this tissue has been obtained yet [19].

Although the term “backfill” is not in universal use, this intra-articular high signal intensity on T1-weighted MRI of the sacroiliac joints has been documented in 38–63% of patients ≤ 45 years old with SpA and has shown high diagnostic value for SpA [15, 19]. One study even suggested that the presence of this finding should overrule the ASAS definition for a “positive MRI” for sacroiliitis suggestive of SpA, even when no concomitant BME is present [20].

### Ankylosis of the sacroiliac joints

**Fig. 3:**
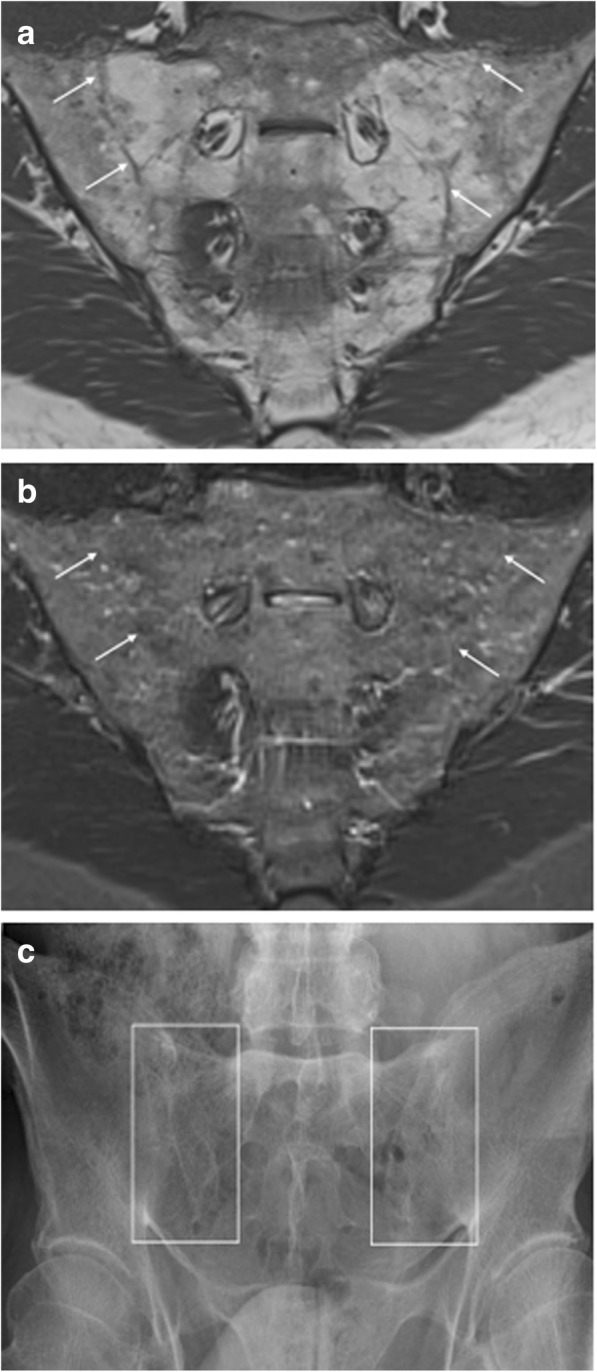
Ankylosis of the sacroiliac joints. Coronal oblique MR images acquired in a 53-year-old man with SpA show bony fusion of the sacroiliac joints as (**a**) high signal intensity on T1-weighted imaging (fatty degeneration) and (**b**) low signal intensity on STIR imaging. A sclerotic remnant of the sacroiliac joints is highlighted (arrows). **c** Radiography of the sacroiliac joints confirms these structural bony changes

Ankylosis of the sacroiliac joints (Fig. 3) is considered a hallmark feature of end-stage axial SpA. This bony bridging may appear as low signal intensity obliteration of articular cortical margins, on most MRI sequences—but it can have high signal intensity on T1-weighted MR images, when the subarticular bone marrow crossing the sacroiliac joint has high-fat content [11, 19, 20].

Similar to intra-articular high signal intensity on T1-weighted MR images, ankylosis is highly specific for SpA, and it has been suggested that its presence should lead to a “positive MRI” for sacroiliitis suggestive of SpA, even in the absence of BME [19, 20, 22].

## MRI features of new bone formation in the spine

**Fig. 4:**
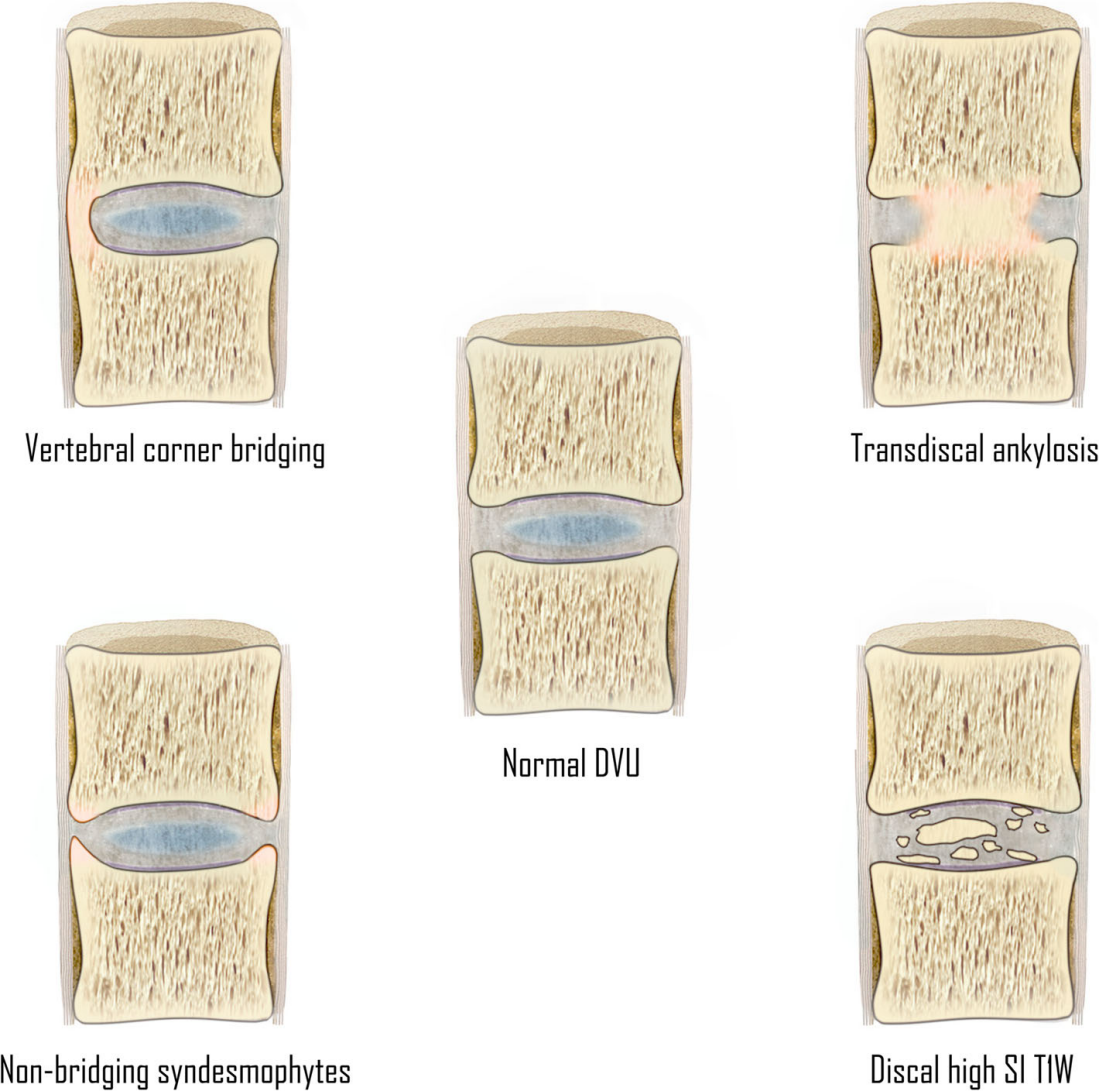
Features associated with new bone formation at the disco-vertebral unit of patients with SpA. (DVU = disco-vertebral unit; SI T1W = signal intensity on T1-weighted MR images)

An illustration of the features associated with new bone formation at the disco-vertebral unit has been presented in Fig. 4.

### Discal high signal intensity on T1-weighted MR images

**Fig. 5:**
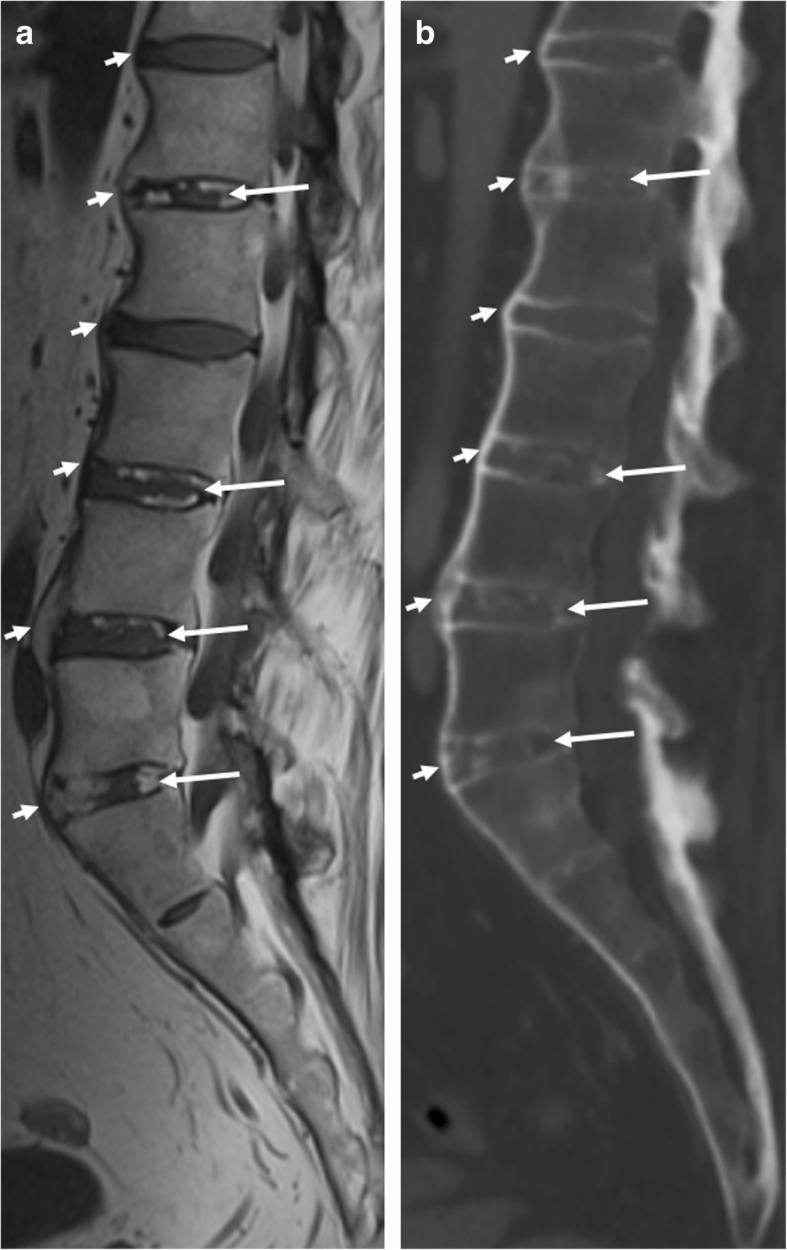
Discal high signal intensity on T1-weighted MR images. **a** Sagittal T1-weighted MR images of the lumbosacral spine acquired in a 45-year-old man with SpA show signal intensity (long arrows) similar to that of adipose tissue within the intervertebral disc. **b** Computed tomography (CT) of the same intervertebral discs show discal calcification (long arrows). Also, notice the discordance in the visibility between CT and MRI for the evaluation of anterior bridging syndesmophytes (short arrows)

Discal high signal intensity on T1-weighted MR images (Fig. 5) has only been examined in a limited number of studies. It has been hypothesized to represent early discal calcification [21, 23–27]. It is defined as the presence of high signal intensity similar to adipose tissue on T1-weighted images within the intervertebral disc, present on two consecutive slices and measuring half of the disc height and a quarter of the vertebra width on at least one slice [21]. In a recent case-control study, discal high T1 signal intensity appeared to be something that was remarkably specific for SpA, although this needs independent validation [21].

It remains a possibility that this signal change of the intervertebral disc could be present in other diseases which also show new bone formation, e.g., diffuse idiopathic skeletal hyperostosis (DISH) [21]. However, no studies concerning this topic have been published to date.

### Non-bridging syndesmophytes

**Fig. 6:**
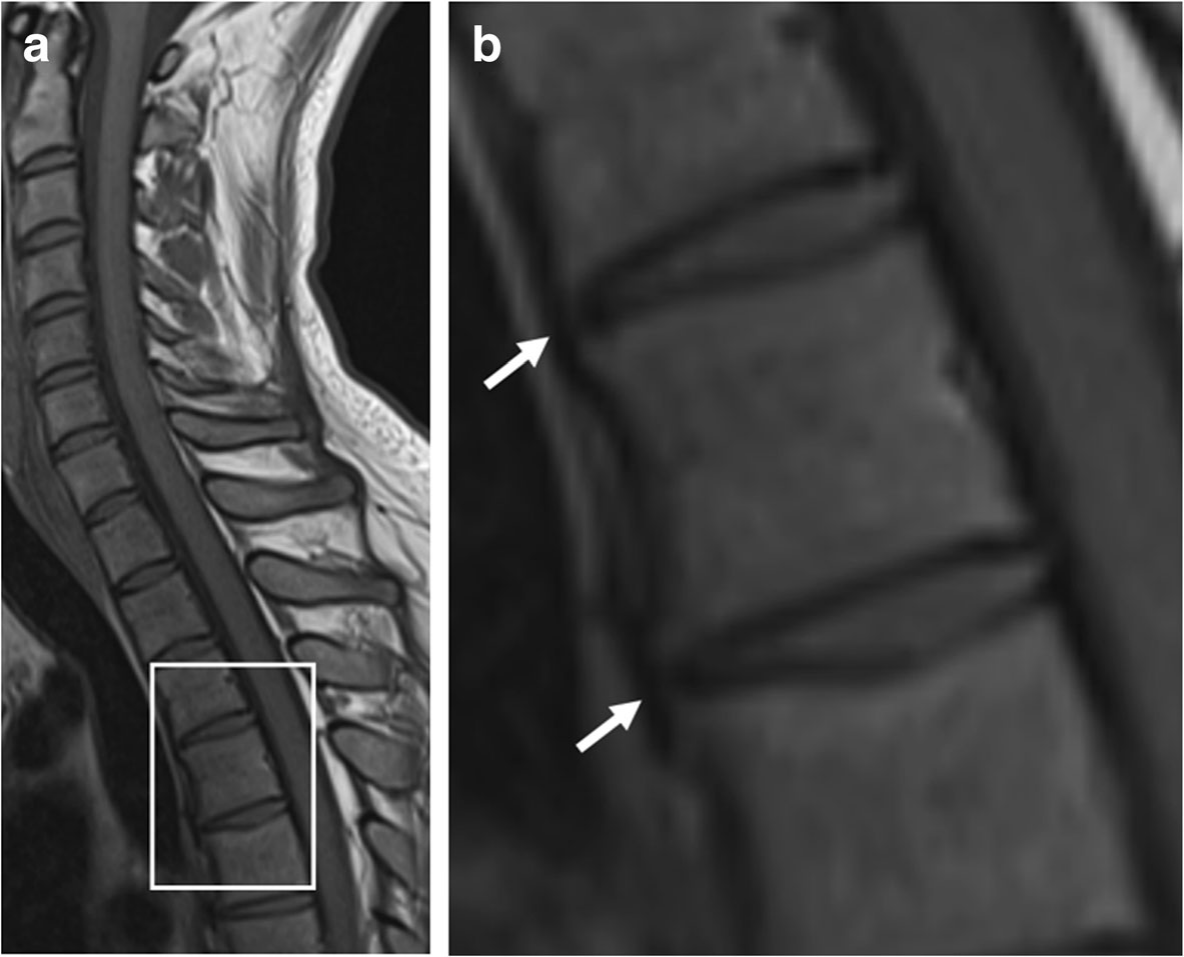
Non-bridging syndesmophytes. Sagittal T1-weighted MR image shows anterior syndesmophytes (arrows) in a patient without SpA. No other disco-vertebral units besides Th3-Th4 and Th4-Th5 show bony changes. Note: **b** is an enlargement of the region outlined in **a**

Syndesmophyte formation (Fig. 6) is defined as bony growth originating from the Sharpey fibers of the annulus fibrosus [11, 13, 21]. On sagittal spinal MRI, syndesmophytes will be observed as longitudinal bony outgrowths at the anterior and posterior corners of the vertebral bodies, oriented craniocaudally. The signal intensity on T1-weighted images is isointense to red bone marrow or hyperintense to red bone marrow—in case of presence of fatty bone marrow [21].

Although a sign of new bone formation, the value of non-bridging syndesmophytes for SpA is questionable on spinal MRI. Firstly, syndesmophytes that are clearly visible radiographically can often hardly be seen on MRI [28]. Secondly, two separate MRI studies demonstrated that non-bridging syndesmophytes are frequently observed in patients without SpA [18, 21]. Finally, non-bridging syndesmophytes in the absence of other spinal features of new bone formation were only seen in patients without SpA [21]. This suggests that non-bridging syndesmophytes on MRI should not be used for purposes of SpA diagnosis. It is also unclear how these relate to disease progression.

### Ankylosis of the vertebral bodies

#### Vertebral corner bridging

**Fig. 7:**
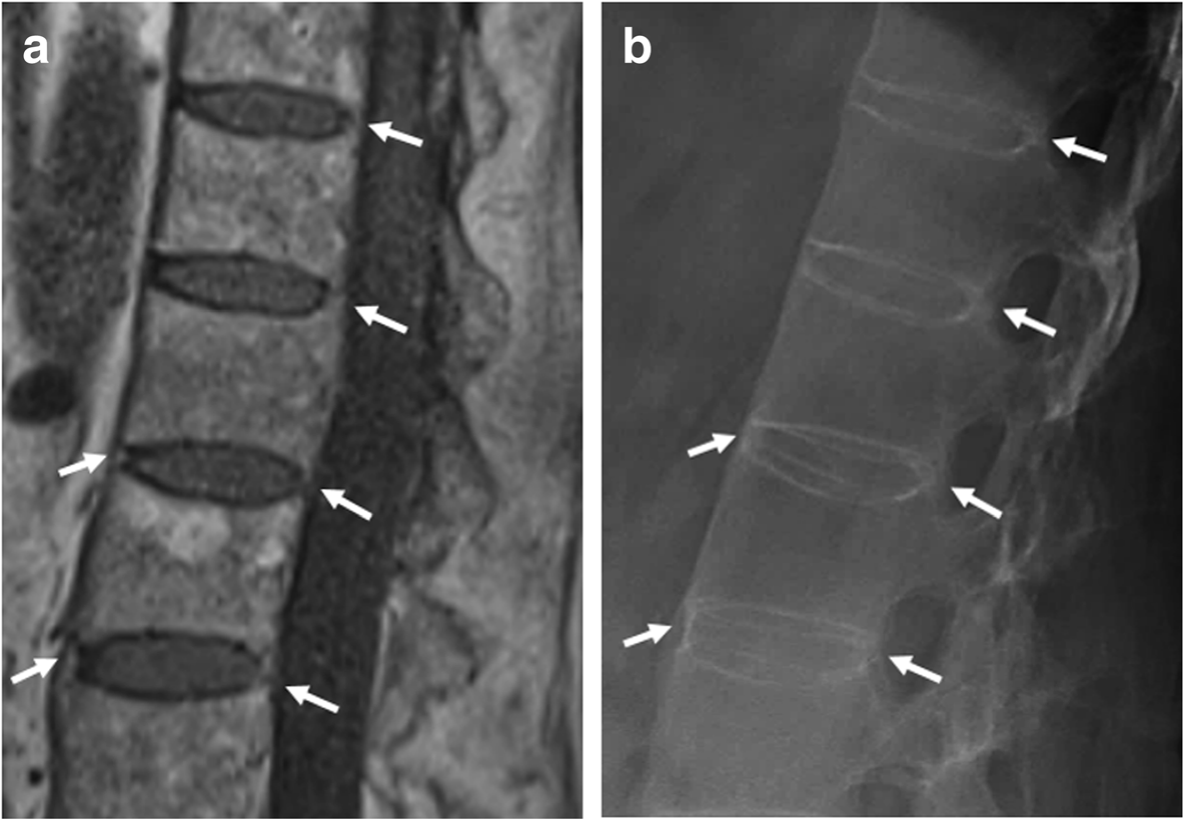
Vertebral corner bridging. **a** Sagittal T1-weighted MR imaging acquired in a 57-year-old man with SpA shows bridging syndesmophytes (arrows), and (**b**) radiography of the same vertebrae confirms these vertebral corner bridges (arrows) resulting in a “bamboo spine” configuration. Note that the smaller anterior syndesmophytes are more clearly visualized on radiograph than MRI, which is typical

Vertebral corner bridging is also referred to as “bridging syndesmophytes” or “ankylosis within the annulus fibrosus” (Fig. 7) [13, 21]. On sagittal spinal MRI, vertebral corner bridging is observed as the bony fusion of the anterior or posterior corners of the vertebral bodies, at the Sharpey fibers of the annulus fibrosus of the intervertebral disc [21]. The signal intensity on T1-weighted images is isointense to red bone marrow or hyperintense to red bone marrow—in case of presence of fatty bone marrow [21]. In contrast to non-bridging syndesmophytes, this MRI feature is specific for SpA and is potentially a reliable indicator of SpA [21].

In extensive cases, vertebral corner bridging is often accompanied by squaring and sclerosis of the anterior aspect of the vertebral body margins resulting in the radiographic feature of a “bamboo spine” [10]. The appearance of a bamboo spine is less evident on MRI, but vertebral corner bridging can clearly be observed [21, 28].

#### Transdiscal ankylosis

**Fig. 8:**
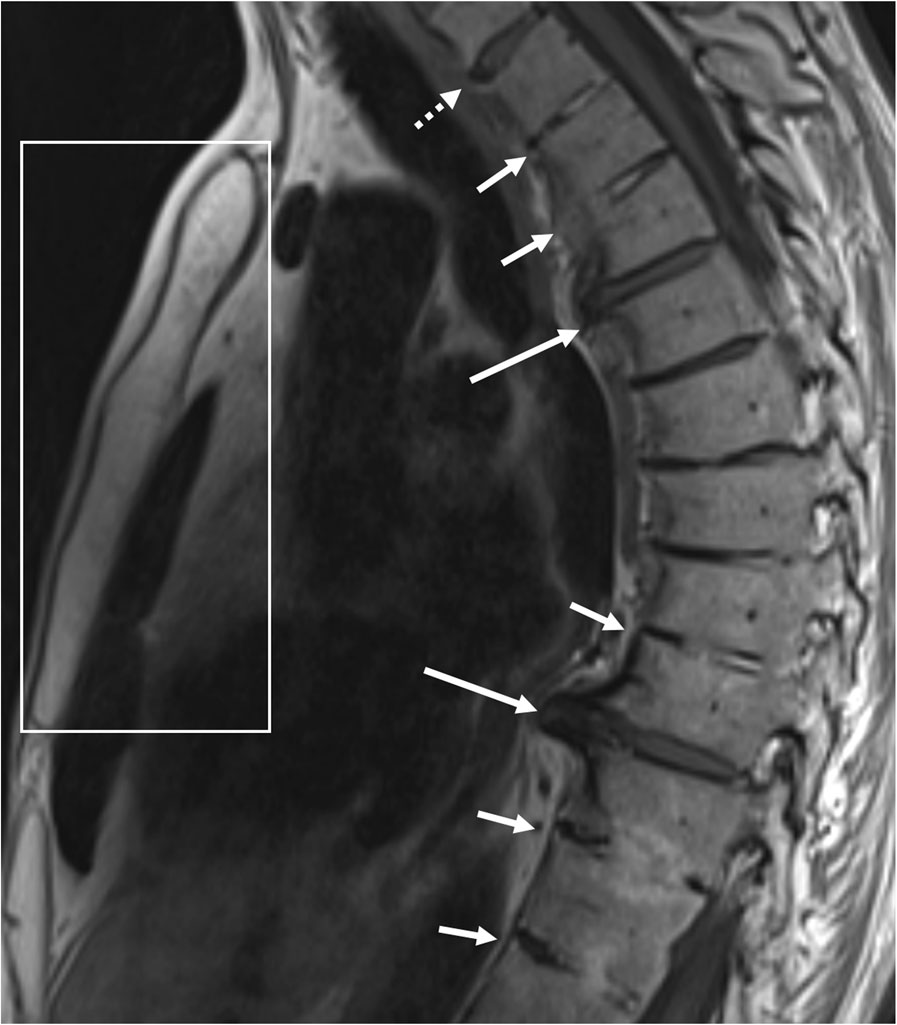
Extensive—and several types of—new bone formation in one patient with SpA. Sagittal T1-weighted MR image shows how bony fusion of the manubriosternal joint can be observed on sagittal imaging of the spine in a routine SpA protocol (rectangle). Also, notice the other types of new bone formation: syndesmophytes (dotted arrow), discal high signal intensity and/ or progressive trandiscal ankylosis (short arrow), and osteophytes (long arrow). This image demonstrates that osteophytes can be seen in patients with SpA at levels otherwise unaffected by SpA, perhaps due to increased mechanical loads on the remaining functional disco-vertebral units

Also referred to as “non-corner ankylosis” [21], this MRI feature (Fig. 8) is defined as bony fusion crossing the vertebral joint space through the expected location of the nucleus pulposus in the intervertebral disc, with obliteration of the cortical margins of the vertebral body [11, 13, 21]. Similar to syndesmophytes, the signal intensity on T1-weighted images is isointense to red bone marrow or hyperintense to red bone marrow—in case of presence of fatty bone marrow [21]. This MRI feature is specific for axial SpA and considered a reliable indicator of SpA [21].

It is generally considered a marker of late disease, as axial SpA almost always starts in the sacroiliac joints, typically leaving the spine unaffected for a longer period of time; however, it can also be found in younger patients with more extensive disease [11, 21].

#### Ankylosis of the intervertebral synovial joints

To the best of our knowledge, no MRI studies regarding the MR features and prevalence of ankylosis of intervertebral synovial joints in SpA have been published. This may be because detailed evaluation of these joints requires coronal or transverse slices, which are time-consuming to obtain at MRI and not generally found in standard SpA MRI protocols [11, 21, 29, 30].

**Fig. 9:**
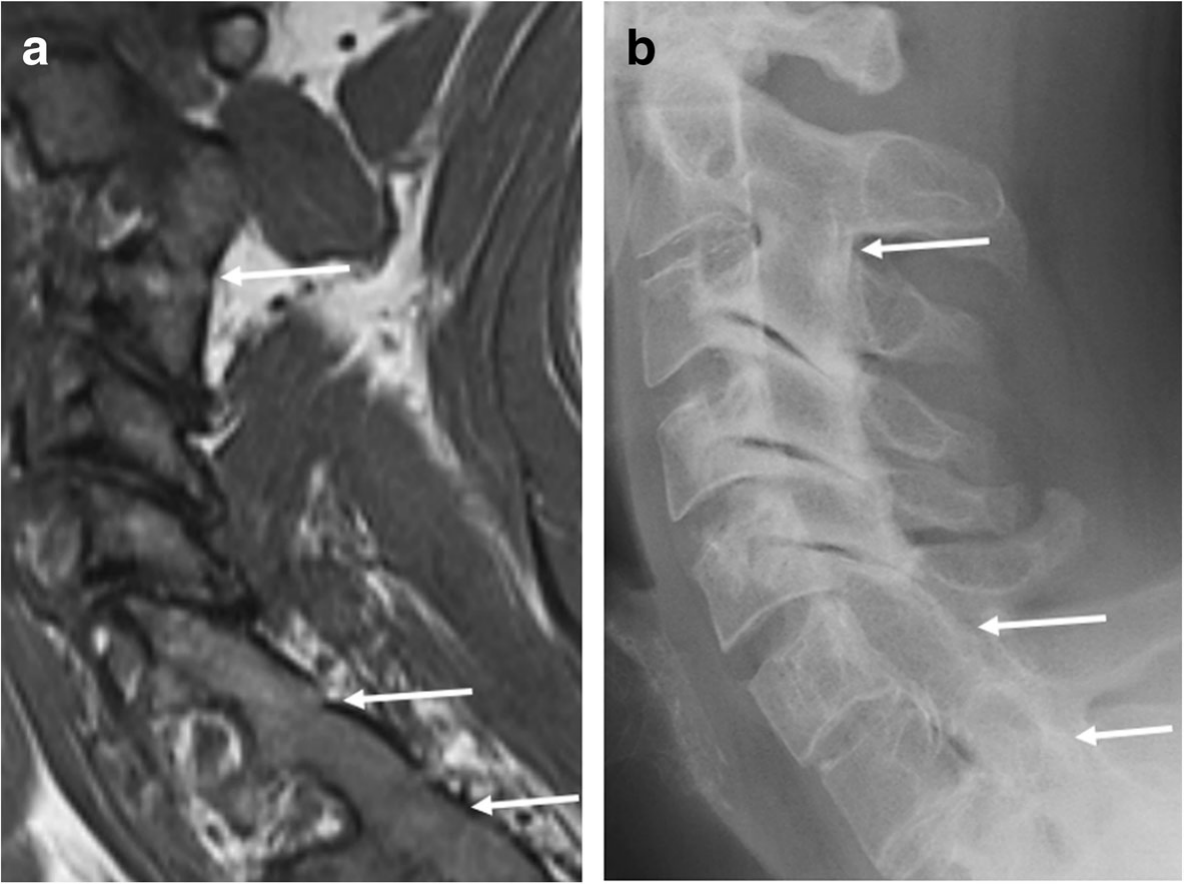
Ankylosis of the facet joints. **a** Sagittal T1-weighted MR image acquired in a 34-year-old man with SpA shows ankylosis of the facet joints of C2-C3, C6-C7, and C7-Th1 (arrows). **b** Radiography of the cervical spine confirms this bony fusion of these joints (arrows)

There are three types of intervertebral synovial joints, i.e., costovertebral, costotransverse, and zygapophyseal (facet) joints. Only the facet joints—to a certain extent—can be assessed for ankylosis on standard sagittal MRI. When visible, they should be assessed for ankylosis (Fig. 9), as it has been suggested that these joints are primarily and early involved in the course of the disease [30, 31].

## MRI features of new bone formation in the sternum

Although rarely discussed—and not even mentioned in the ASAS handbook—ankylosis of the manubriosternal joint (Fig. 8) or the sternoclavicular joints can occur in SpA [11, 32]. Nevertheless, sagittal imaging planes—as obtained in standard MRI of the spine in SpA—allow detailed evaluation of the manubriosternal joint. Therefore, it is important that no saturation bands are placed over the sternum in standard MR imaging of the spine for SpA, as this feature of new bone formation might be missed. Note that coronal planes can also be useful for assessing the sternum, but axial planes are not.

## Pitfalls in MR imaging diagnosis

### Diffuse idiopathic skeletal hyperostosis

**Fig. 10:**
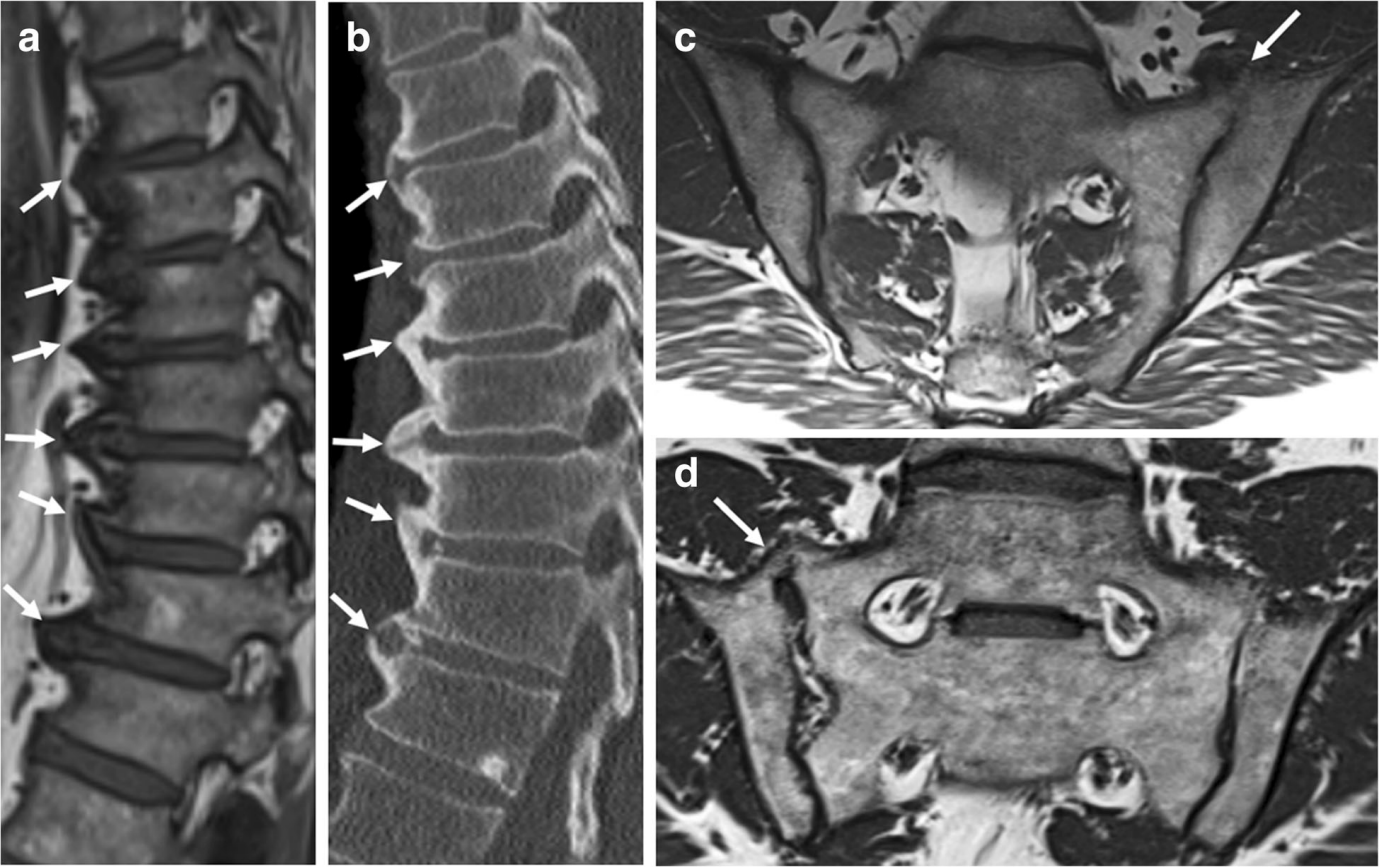
Pitfalls in imaging and diagnosis of new bone formation: DISH. **a** Sagittal T1-weighted MR image and (**b**) computed tomography (CT) show hyperproliferative ossification of the anterior longitudinal ligament, resulting in bulky horizontally oriented osteophytes (arrows) with—in this case—an average growth angle of > 45° from vertical. **c**, **d** Semicoronal T1-weighted MR images show bridging ossification (arrows) at the anterior and superior aspect of the sacroiliac joints

Diffuse idiopathic skeletal hyperostosis (DISH) is also referred to as “Forestier disease” (Fig. 10). DISH patients typically have bulky osteophytes, which often exceed the length of anterior longitudinal ligament [33]. The Resnick criteria are helpful for the diagnosis of DISH: hyperproliferative bony changes in ≥ 4 adjacent vertebrae, preservation of the intervertebral disc space, and absence of apophyseal joint or inflammatory sacroiliac changes [34, 35]. If still ambiguous, it has also been suggested to evaluate the growth angle of the vertebral edge to differentiate syndesmophytes (primarily craniocaudal orientation ≤ 45° from vertical) from spondylophytes (primarily horizontal, > 45° from vertical) [36].

Although DISH is most typically seen on the right side of the thoracic spine, it can also be present in the sacroiliac joints [11]. Furthermore, recently, it has been shown that sacroiliac fusion, anterior and posterior bridging, and entheseal bridging also occur significantly in DISH [37].

### Congenital vertebral fusion

**Fig. 11:**
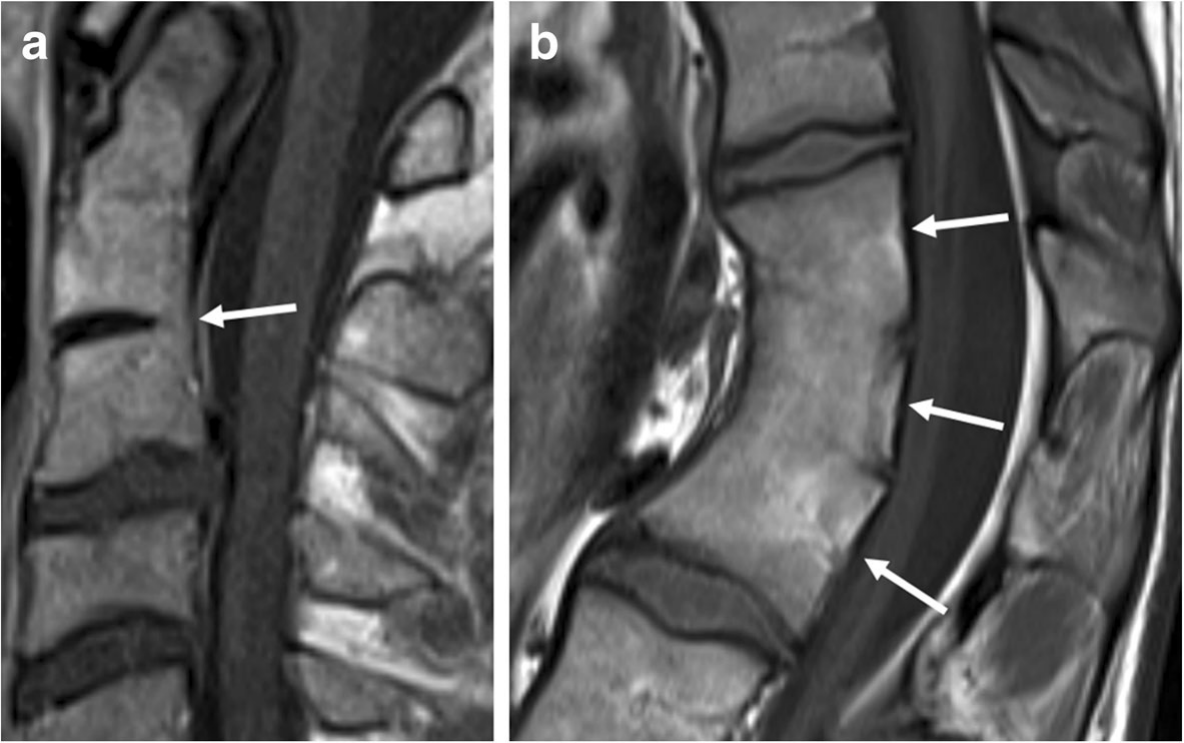
Pitfalls in imaging and diagnosis of new bone formation: congenital block vertebrae. Sagittal T1-weighted MR images show (**a**) partial congenital block vertebra of C2-C3 (arrow) and (**b**) complete congenital block vertebra consisting out of three vertebrae (arrows). Note the narrowed antero-posterior diameter in **b**, a typical sign of complete congenital fusion. Also, note that the height in **b** is less than the expected sum of three vertebrae and two intervertebral discs

Also referred to as “congenital block vertebrae” (Fig. 11), this vertebral fusion is due to a failure in the process of segmentation during the fetal period. Fusion of the vertebrae can be partial or complete, dependent on the involvement of anterior and/or posterior elements. At the level of the intervertebral disc, there is often a “waist.” The height of a block vertebra should be that of the two vertebrae and the intervertebral disc [38]; however, this is not always the case in clinical practice—as demonstrated in Fig. 11b. Since vertebrae grow in antero-posterior diameter during childhood, fusion that is congenital or developmental is often associated with narrow antero-posterior width of the affected vertebrae, a clue that can distinguish this from fusion later in life.

### Acquired vertebral fusion

**Fig. 12:**
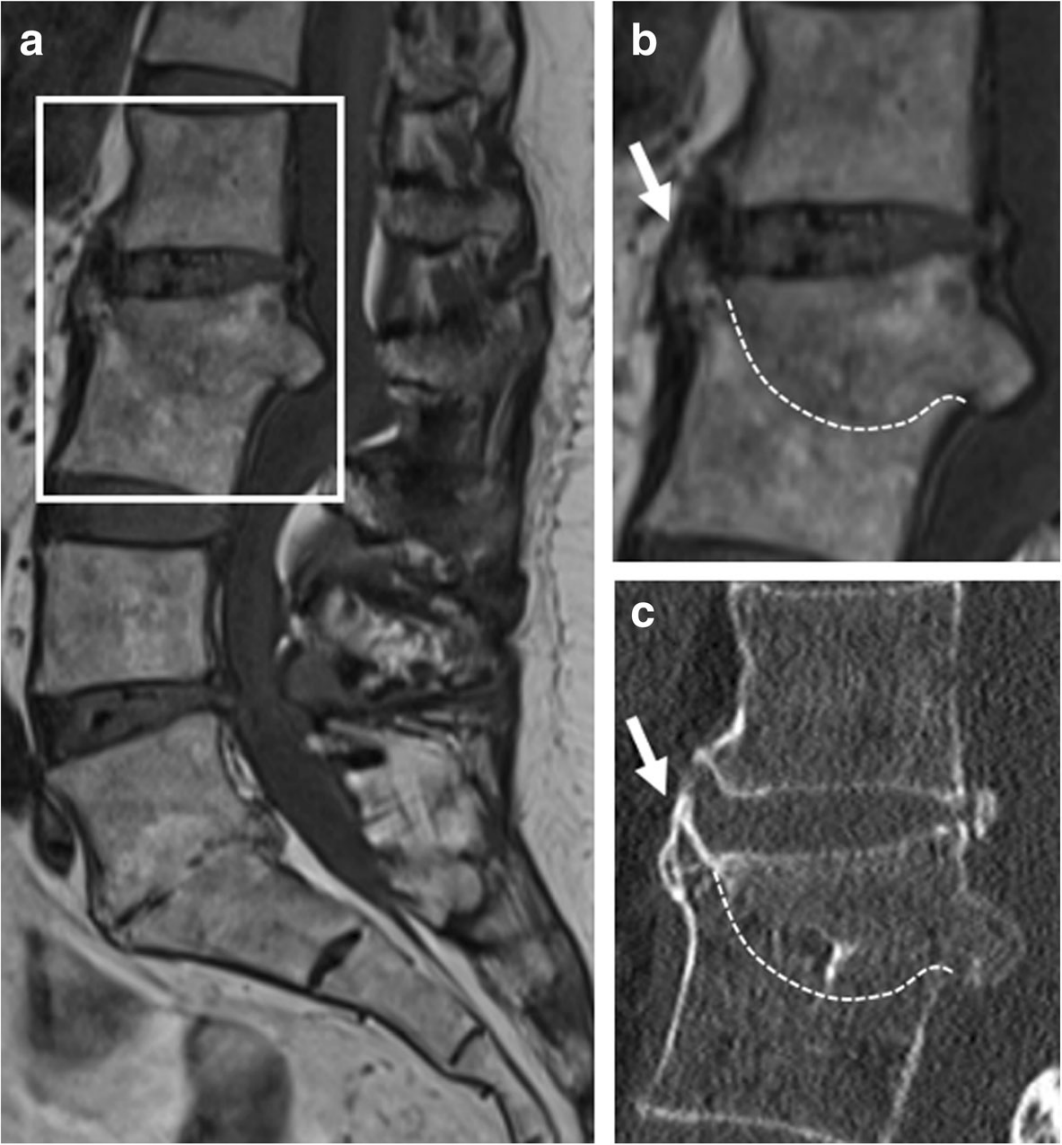
Pitfalls in imaging and diagnosis of new bone formation: sequelae of infectious spondylodiscitis. **a**, **b** Sagittal T1-weighted MR images and (**c**) computed tomography (CT) show vertebral fusion (dotted line) of L2-L3 after destruction of the vertebrae and intervertebral disc due to tuberculous spondylodiscitis. Note that the protrusion of the remainder of the anterior corner of the L3 mimics plump syndesmophyte formation (short arrow). L5-S1 also shows signs of vertebral fusion after destruction of the intervertebral disc, mimicking transdiscal ankylosis as seen in SpA

**Fig. 13:**
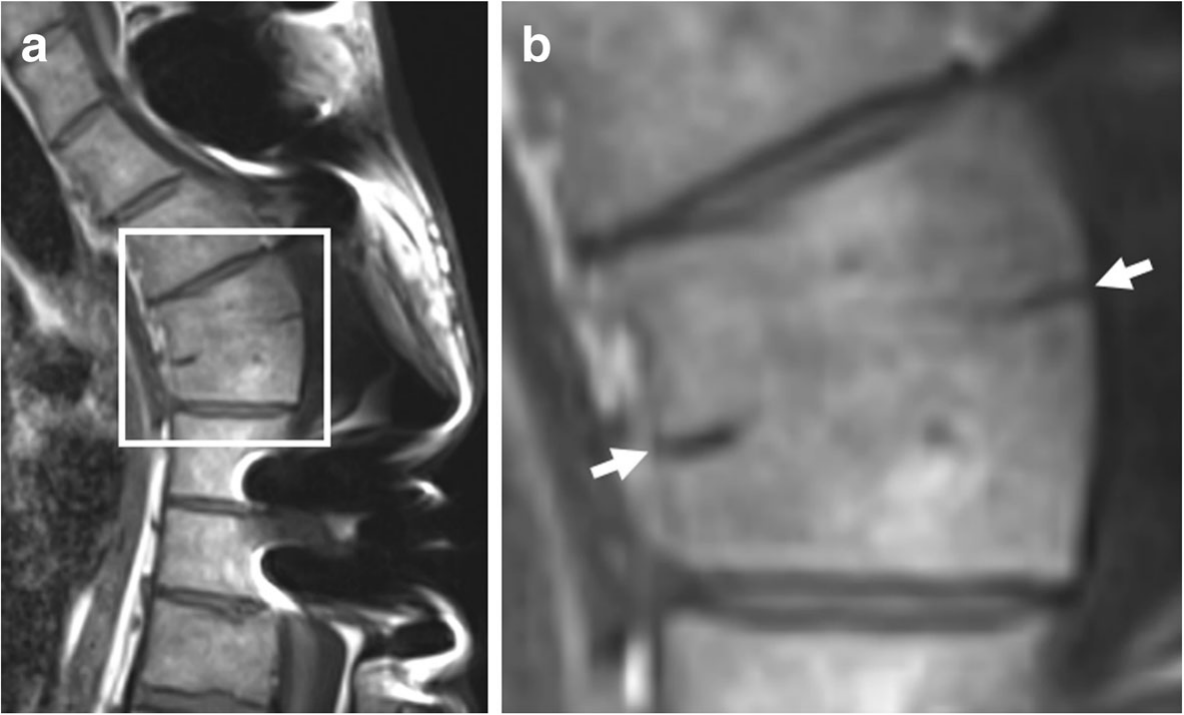
Pitfalls in imaging and diagnosis of new bone formation: post-traumatic vertebral fusion. Sagittal T1-weighted MR images show interbody vertebral fusion years after severe spinal trauma, with discrete remnants of the intervertebral disc (arrows). This image suggests that the original injury included both vertebral endplates and the intervertebral disc. Also note that the height is less than the sum of the two vertebral bodies and the intervertebral disc, and antero-posterior diameter is not narrowed, two features which help differentiate this image from a congenital block vertebra. Note: **b** is an enlargement of the region outlined in **a**

In acquired vertebral fusion, unlike congenital vertebral fusion, the height should be less than the sum of the two vertebral bodies and the intervertebral disc [38]. Acquired intervertebral fusion can occur as a late complication of infectious spondylodiscitis (e.g., Pott’s disease) after 12–24 months (Fig. 12) [39]. Post-traumatic interbody fusion is a rare phenomenon, and it has been suggested that it can only occur when both the opposing endplates and the intervertebral disc are involved in the injury (Fig. 13) [40]. Acquired vertebral fusion is desirable when surgical spinal arthrodesis is performed; however, this is beyond the scope of this review.

## Conclusions

This review demonstrates the most important MRI features of new bone formation in the axial skeleton of patients with SpA for daily clinical practice. When reading MR of the sacroiliac joints, it is important to examine the joint space on T1-weighted images for high signal intensity (“backfill”) or ankylosis, as these signs are very specific for SpA. When reading MR of the spine, examine the intervertebral joint space and disc on T1-weighted images for discal high signal intensity or the presence of ankylosis (i.e., vertebral corner bridging or transdiscal ankylosis) as these signs are also very specific for SpA. When reporting non-bridging syndesmophytes on MRI of the spine, keep in mind that this finding at MRI is neither sensitive nor specific for SpA. When reading MR of the spine, inspection of the facet joints and manubriosternal joint can reveal under-appreciated features of SpA.

It is important for the radiologist to keep in mind that, although new bone formation is a hallmark feature of SpA, there are some pitfalls: DISH, sequelae of infectious spondylodiscitis, congenital block vertebra, or post-infectious or post-operative vertebral fusion also show or mimic new bone formation.

Although MRI features of new bone formation are not included in current ASAS classification criteria, they should be specifically evaluated to obtain a complete assessment of the spine and sacroiliac joints in a patient who may have SpA. These findings can provide an additional qualitative aspect to the presence of bone marrow edema and can be helpful when the outcome of an MRI is ambiguous or to evaluate disease progression.

## Data Availability

Not applicable.

## Abbreviations

ASAS: Assessment of SpondyloArthritis international Society
DISH: Diffuse idiopathic skeletal hyperostosis
SpA: Spondyloarthritis

## References

[1] Braun J, Sieper J (2007). Ankylosing spondylitis. Lancet.

[2] Ward MM, Deodhar A, Akl EA (2016). American College Of Rheumatology/Spondylitis Association of America/Spondyloarthritis research and treatment network 2015 recommendations for the treatment of ankylosing spondylitis and non-radiographic axial spondyloarthritis. Arthritis Rheumatol.

[3] Lacout A, Rousselin B, Pelage J (2008). CT and MRI of spine and sacroiliac involvement in spondylarthropathy. AJR Am J Roentgenol.

[4] Dougados M, Baeten D (2011). Spondyloarthritis. Lancet.

[5] Jans L, Van Langenhove C, Van Praet L (2014). Diagnostic value of pelvic enthesitis on MRI of the sacroiliac joints in spondyloarthritis. Eur Radiol.

[6] Baraliakos X, Listing J, von der Recke A, Braun J (2009). The natural course of radiographic progression in ankylosing spondylitis—evidence for major individual variations in a large proportion of patients. J Rheumatol.

[7] Lories RJ, Schett G (2012). Pathophysiology of new bone formation and ankylosis in spondyloarthritis. Rheum Dis Clin North Am.

[8] Sieper J, Appel H, Braun J, Rudwaleit M (2008). Critical appraisal of assessment of structural damage in ankylosing spondylitis: implications for treatment outcomes. Arthritis Rheum.

[9] Lories RJ, Luyten FP, de Vlam K (2009). Progress in spondylarthritis. Mechanisms of new bone formation in spondyloarthritis. Arthritis Res Ther.

[10] Poddubnyy D, Sieper J (2017). Mechanism of new bone formation in axial spondyloarthritis. Curr Rheumatol Rep.

[11] Sieper J, Rudwaleit M, Baraliakos X (2009). The Assessment of Spondyloarthritis International Society (ASAS) handbook: a guide to assess spondyloarthritis. Ann Rheum Dis.

[12] Lambert RG, Bakker PA, van der Heijde D (2016). Defining active sacroiliitis on MRI for classification of axial spondyloarthritis: update by the ASAS MRI working group. Ann Rheum Dis.

[13] Hermann KG, Baraliakos X, van der Heijde DM (2012). Descriptions of spinal MRI lesions and definition of a positive MRI of the spine in axial spondyloarthritis: a consensual approach by the ASAS/OMERACT MRI study group. Ann Rheum Dis.

[14] Aydingoz U, Yildiz AE, Ozdemir ZM, Yildirim SA, Erkus F, Ergen FB (2012). A critical overview of the imaging arm of the ASAS criteria for diagnosing axial spondyloarthritis: what the radiologist should know. Diagn Interv Radiol.

[15] Weber Ulrich, Pedersen Susanne J, Ostergaard Mikkel, Rufibach Kaspar, Lambert Robert G, Maksymowych Walter P (2012). Can erosions on MRI of the sacroiliac joints be reliably detected in patients with ankylosing spondylitis? A cross-sectional study. Arthritis Research & Therapy.

[16] Maksymowych WP, Wichuk S, Chiowchanwisawakit P, Lambert RG, Pedersen SJ (2014). Fat metaplasia and backfill are key intermediaries in the development of sacroiliac joint ankylosis in patients with ankylosing spondylitis. Arthritis Rheumatol.

[17] Maksymowych WP, Wichuk S, Chiowchanwisawakit P, Lambert RG, Pedersen SJ (2015). Development and preliminary validation of the spondyloarthritis research consortium of Canada magnetic resonance imaging sacroiliac joint structural score. J Rheumatol.

[18] de Hooge M, van den Berg R, Navarro-Compán V (2016). Patients with chronic back pain of short duration from the SPACE cohort: which MRI structural lesions in the sacroiliac joints and inflammatory and structural lesions in the spine are most specific for axial spondyloarthritis?. Ann Rheum Dis.

[19] Laloo F, Herregods N, Varkas G (2017). MR signal in the sacroiliac joint space in spondyloarthritis: a new sign. Eur Radiol.

[20] Laloo F, Herregods N, Jaremko JL, Verstraete K, Jans L (2018). MRI of the sacroiliac joints in spondyloarthritis: the added value of intra-articular signal changes for a ‘positive MRI’. Skeletal Radiol.

[21] Laloo F, Herregods N, Jaremko JL (2019). New bone formation in the intervertebral joint space in spondyloarthritis: an MRI study. Eur J Radiol.

[22] Jans L, Coeman L, Van Praet L (2014). How sensitive and specific are MRI features of sacroiliitis for diagnosis of spondyloarthritis in patients with inflammatory back pain?. JBR-BTR.

[23] Major NM, Helms CA, Genant HK (1993). Calcification demonstrated as high signal intensity on T1-weighted MR images of the disks of the lumbar spine. Radiology.

[24] Vignaux O, Sarrazin JL, Cordoliani YS, Cosnard G (1994) Hypersignal of the intervertebral disks in T1-weighted spin-echo MRI sequences. J Radiol 75:363–367 8083851

[25] Bangert BA, Modic MT, Ross JS (1995). Hyperintense disks on T1-weighted MR images: correlation with calcification. Radiology.

[26] Tyrrell PN, Davies AM, Evans N, Jubb RW (1995). Signal changes in the intervertebral discs on MRI of the thoracolumbar spine in ankylosing spondylitis. Clin Radiol.

[27] Malghem J, Lecouvet FE, François R (2005). High signal intensity of intervertebral calcified disks on T1-weighted MR images resulting from fat content. Skeletal Radiol.

[28] Braun J, Baraliakos X, Golder W (2004). Analysing chronic spinal changes in ankylosing spondylitis: a systematic comparison of conventional x rays with magnetic resonance imaging using established and new scoring systems. Ann Rheum Dis.

[29] Hermann KG, Althoff CE, Schneider U (2005). Spinal changes in patients with spondyloarthritis: comparison of MR imaging and radiographic appearances. Radiographics.

[30] Baraliakos X (2017). Imaging in axial spondyloarthritis. Isr Med Assoc J.

[31] de Vlam K, Mielants H, Veys EM (1999). Involvement of the zygapophyseal joint in ankylosing spondylitis: relation to the bridging syndesmophyte. J Rheumatol.

[32] Ehara S (2010). Manubriosternal joint: imaging features of normal anatomy and arthritis. Jpn J Radiol.

[33] Baraliakos X, Listing J, Buschmann J, von der Recke A, Braun J (2012). A comparison of new bone formation in patients with ankylosing spondylitis and patients with diffuse idiopathic skeletal hyperostosis: a retrospective cohort study over six years. Arthritis Rheum.

[34] Resnick D, Shaul SR, Robins JM (1975). Diffuse idiopathic skeletal hyperostosis (DISH): Forestier’s disease with extraspinal manifestations. Radiology.

[35] Oudkerk SF, de Jong PA, Attrach M (2017). Diagnosis of diffuse idiopathic skeletal hyperostosis with chest computed tomography: inter-observer agreement. Eur Radiol.

[36] Baraliakos X, Listing J, Rudwaleit M (2007). Progression of radiographic damage in patients with ankylosing spondylitis: defining the central role of syndesmophytes. Ann Rheum Dis.

[37] Leibushor N, Slonimsky E, Aharoni D, Lidar M, Eshed I (2017). CT abnormalities in the sacroiliac joints of patients with diffuse idiopathic skeletal hyperostosis. AJR Am J Roentgenol.

[38] Kumar R, Guinto FC, Madewell JE, Swischuk LE, David R (1988). The vertebral body: radiographic configurations in various congenital and acquired disorders. Radiographics.

[39] Batirel A, Erdem H, Sengoz G (2015). The course of spinal tuberculosis (Pott disease): results of the multinational, multicentre Backbone-2 study. Clin Microbiol Infect.

[40] Korres DS, Babis GC, Paraskevakou H, Stamos K, Tsarouchas J, Lykomitros V (2000). Spontaneous interbody fusion after controlled injuries to the spine: an experimental study in rabbits. J Spinal Disord.

